# Foxo3a Expression Is a Prognostic Marker in Breast Cancer

**DOI:** 10.1371/journal.pone.0070746

**Published:** 2013-08-13

**Authors:** Ying Jiang, Lin Zou, Wei-Qi Lu, Yong Zhang, Ai-Guo Shen

**Affiliations:** 1 Department of General Surgery, Zhongshan Hospital, Fudan University, Shanghai, P.R. China; 2 Department of General Surgery, Affiliated Hospital of Nantong University, Nantong, Jiangsu, P.R. China; 3 Department of Pathology, The First People's Hospital of Yancheng affiliated with Nantong University, Yancheng, Jiangsu, P.R. China; 4 Department of Pathology, Nantong University, Nantong, P.R. China; Health Canada and University of Ottawa, Canada

## Abstract

The forkhead box transcription factor Foxo3a has been implicated to play a critical role in various cancers by suppressing tumor growth. Recent studies have identified Foxo3a as a key regulator of Estrogen Receptor-α (ERα). In the present study, we examined the expression of Foxo3a, and investigated its clinical significance and correlation with ER and prognostic role in patients with breast cancer. Immunohistochemical analysis was performed on tumors from 70 breast cancer patients. Interpretable Foxo3a expression was analyzed along with major clinicopathologic variables, and a comparison was made with corresponding 5-year clinical follow-up data. Foxo3a protein expression correlated with ER positivity (P<0.001), histologic grade (1, 2) (P = 0.002), axillary lymph node negativity (P<0.001) and TNM stage (1, 2) (P<0.001). Moreover, the Kaplan-Meier survival curves of the study population showed that a high expression level of Foxo3a was significantly correlated with long-term survival (P<0.0001). In a multivariate analysis, Foxo3a expression was identified as a favorable independent prognostic factor in overall survival (P = 0.038). In conclusion, our results indicated that Foxo3a expression is a favorable prognostic marker in breast cancer. In addition, Foxo3a staining could potentially be used in patient stratification in conjunction with other prognostic markers.

## Introduction

Worldwide, breast cancer is the second most prevalent malignancy after lung cancer and the fifth most common cause of cancer death; it is the disease women fear most. Despite advances in early diagnosis and therapy, more than 44,000 women in the United States will die of metastatic disease each year [1]. Although progress has been made in the management of breast cancer patients, the mechanism underlying the development of this heterogeneous disease remains largely unclear, and the genetic and molecular alterations in breast cancer are not fully understood. This has motivated considerable efforts toward finding novel, clinically efficient, and readily available prognostic or predictive markers of breast cancer.

Members of the FOXO family of forkhead transcription factors are critical positive regulators of longevity in species as diverse as worms and flies [2]–[4]. The FOXO subfamily of forkhead transcription factors, FOXO1 (FKHR), FOXO3a (FKHRL1), and FOXO4 (AFX), is regulated by the PI3K/Akt pathway. FOXO proteins have been implicated in the control of genes involved in multiple cellular processes, including the cell cycle [5], [6], cell death [7], [8], neoplastic transformation [9]–[11], epithelial-to-mesenchymal transition [12], longevity [13], [14], metabolism [15], [16], and protection from oxidative stress [17]–[19]. FOXOs are phosphorylated by Akt on highly conserved serine and threonine residues, resulting in impaired DNA binding activity and increased binding to the chaperone protein 14-3-3. Newly formed 14-3-3-FOXO complexes are then exported from the nucleus [20], thereby inhibiting the FOXO-dependent transcription of key target genes that promote cell cycle arrest and apoptosis, such as p27^Kip1^ and Bim [6], [21]–[23]. Thus, the inactivation of FOXOs controls diverse functions, including cell differentiation, proliferation, cell death, metabolism, and longevity [24]. In brief, FOXOs play a complex role in tumorigenesis [25].

Estrogen receptors (ERs) play key roles in the growth and development of human breast tumors through their mitogenic effects on breast cancer cells. This concept led to the development of selective estrogen receptor (ER) modulators, such as tamoxifen and toremifene, as endocrine therapy for breast cancer [26]. These modulators bind to estrogen receptor alpha (ERa), an estrogen-dependent transcriptional factor, and thereby regulate growth, development, differentiation, and homeostasis by binding to EREs in DNA to modulate the transcription of target genes [27]. A previous study has shown that ERa is expressed in 10% to 15% of luminal epithelial cells in normal breast tissue, and these cells are generally considered slowly proliferating and well-differentiated cells types [28]. However, >50% of breast cancers express ERa at the time of initial diagnosis [29]. Thus, ERa has provided an exploitable target for therapy.

From a clinical view, the presence of ERa in breast cancer is viewed as a favorable prognostic indicator because it is linked to a lower risk of relapse and better overall disease-free survival [30]. However, only approximately 50% of ER-positive tumors respond to currently available hormonal therapies, and most tumors that initially respond eventually become resistant to endocrine therapy, even though ER may still be present in the tumor tissue [31]. Thus, to prevent or reverse anti-estrogen resistance, the signaling mechanisms underlying the regulation of ER function need to be explored. Currently, FOXO3a is receiving considerable attention with respect to ERa function because it can interact with forkhead box M1 (FOXM1) on the ERα promoter and regulate ERα expression [32]–[34]. Using an orthotropic breast tumor animal model, Zou et al. showed that FOXO3a suppressed E2-induced tumorigenesis in MCF-7 cells, suggesting that FOXO3a has a critical tumor suppression role in estrogen-dependent breast cancer [35]. Although previous studies have shown a functional interaction between FOXO3a and ERα, there is no research on the clinical significance of the expression and association of these two proteins in human breast carcinomas. In the present study, we examined the expression of FOXO3a by immunohistochemical analysis in breast carcinoma specimens of 70 patients and compared FOXO3a expression with various established disease markers, such as tumor size, histologic grade, axillary lymph node status, ER status, PR status, HER-2 status, TNM stage and histology, and then performed survival analyses, including standard prognostic variables, in female patients with breast cancer.

## Materials and Methods

### Tissue Samples

70 breast cancer and 20 benign breast disease specimens from patients who underwent surgery between 2001 and 2003 at the Department of Pathology, Affiliated Hospital of Nantong University, were formalin fixed and paraffin embedded for histopathologic and immunohistochemical analyses. All tumors were from patients with newly diagnosed breast cancer who had received no therapy before sample collection. The TNM system of tumor staging and histological grading were performed according to the guidelines from the World Health Organization guidelines [36]. The samples were frozen in liquid nitrogen immediately after surgical removal and maintained at −80°C until use in Western blot analysis. All human tissue was collected using protocols approved by the Ethics Committee of the Affiliated Hospital of Nantong University.

### Antibody

Primary antibodies included rabbit anti-human Foxo3a antibody (Cell Signaling Technology, Boston, Massachusetts, USA)at a dilution of 1∶100 for Foxo3a, monoclonal mouse anti-human ER antibody (DAKO, Glostrup, Denmark)at 1∶100 dilution for ER, monoclonal mouse anti-human PR antibody (DAKO, Glostrup, Denmark) at 1∶100 dilution for PR, and rabbit anti-human HER2 antibody(DAKO, Glostrup, Denmark) at 1∶200 dilution for HER2. The rabbit anti-human Foxo3a antibody (Cell Signaling Technology, Boston, Massachusetts, USA) was used at a dilution of 1∶1000 for Western blot analysis according to the manufacturer's instructions.

### Immunohistochemistry

Serial sections that were 4 µm thick were mounted on glass slides coated with 10% polylysine. The sections were dewaxed in xylene and rehydrated in graded ethanol. The endogenous peroxidase activity was blocked by immersion in 0.3% methanolic peroxide for 40 minutes. Immunoreactivity was enhanced by microwaving the tissue sections for 10 minutes in 0.1 M citrate buffer. Immunostaining was performed using the avidin-biotin-peroxidase complex method, and antigen-antibody reactions were visualized with the chromogen diaminobenzidine.

### Immunohistochemical Evaluation

Three independent pathologists (GSS, JGZ and MMC) evaluated the immunostaining results. The Foxo3a-positive cells were counted by monitoring at least 300 cells from at least 10 randomly selected fields. We then calculated the percentage of antigen-positive cells among the total number of cells counted to obtain the labeling index (LI). To allow for univariate and multivariate analyses, we divided the samples into two groups according to the average LI (%): a high expression group (LI≥31.7%) and a low expression group (LI<31.7%). ER and PR expression was assessed by evaluating the proportion and intensity of positively stained carcinoma cells. A score was assigned to represent the estimated percentage of positively stained carcinoma cells as follows:0 = none; 1 = 1%; 2 = 1–10%; 3  = 10–33%; 4 = 33–67%; 5≥67%. An intensity score was assigned to represent the average estimated intensity of staining in positive carcinoma cells as follows: 0 = none, 1 = weak,2 = intermediate, 3 = strong. The proportion score and intensity score were added to obtain a total score ranging from 0 to 8 [37]. The immunohistochemistry results were classified on the basis of the total scores, with 0 to 2 classified as −, 3 to 4 classified as 1+, 5 to 6 classified as 2+ and 5 to 6 classified as 3+.To determine the score of HER2 expression, the membrane-staining pattern was estimated and scored on a scale of − to 3+. Tumors with a score ≥2+ were considered to be positive for HER2 overexpression.

### Cell Culture

One normal human breast epithelial cell line HBL-100 and three human breast cancer cell lines (MCF-7, MDA-MB-231 and MDA-MB-435), which were a gift from the Department of Oncology, Cancer Hospital of Fudan University, were used in this study. All cell lines were maintained in RPMI 1640 (Gibco BRL, Grand Island, NY, USA) supplemented with 10% heat-inactivated fetal calf serum, 2 mM L-glutamine, and 100 U/mL penicillin-streptomycin mixture (Gibco BRL, Grand Island, NY, USA) at 37°C and 5% CO_2_.

### Western Blot Analysis

The cells were washed three times with ice-cold PBS and resuspended in 2× lysis buffer (50 mM Tris-HCl, 120 mM NaCl, 0.5% Nonidet P-40, 100 mM NaF, 200 µM Na_3_VO_4_, and protease inhibitor mixture) and the frozen tissues were homogenized in lysis buffer (1% NP-40, 50 mmol/l Tris, pH 7.5, 5 mmol/l EDTA, 1% SDS, 1% sodium deoxycholate, 1% Triton X-100, 1 mmol/l PMSF, 10 mg/mL aprotinin, and 1 mg/mL leupeptin) and then incubated for 20 min at 4°C with agitation. The lysates were clarified by centrifugation (10 min×12,000 rpm, 4°C). Fifty µg of total protein was resolved by SDS-PAGE and transferred onto polyvinylidene difluoride membranes (Immobilon, Millipore). The membranes were first blocked with 5% nonfat dry milk and then incubated with the primary antibody for 2 hours at room temperature. After the filters were washed three times, they were incubated with horseradish peroxidase-conjugated secondary human anti-mouse or anti-rabbit antibodies (1∶1000; Pierce) for 1 hour at room temperature according to the manufacturer's instructions. The immunocomplexes were detected with an enhanced chemiluminescence system (NEN Life Science Products, Boston, MA).

### Statistical Analysis

The results for continuous variables were presented as the mean±standard deviation. In addition, the statistical significance of the means was calculated using Student's t-test. The Mann-Whitney test was used to examine the association of Foxo3a with the clinicopathological parameters. The nonparametric Spearman's rank correlation coefficient was applied to evaluate the strength of the relationship between Foxo3a and ER expression. Survival analysis was performed using the Kaplan-Meier method, and the curves were compared using the log-rank test. Cox's proportional hazards model was used for univariate and multivariate analyses of the prognostic values. All significance tests were 2 tailed. P<0.05 was considered statistically significant. All computations were carried out using the SPSS 15.0 statistical program.

### Densitometry Analyses

The density of specific bands was measured with a computer-assisted image analysis system (Adobe Systems, San Jose, CA, USA) and normalized against the density of β-actin, and the relative differences between the cell lines were calculated and expressed as relative increases, with the control set as 1. The values were from at least 3 independent reactions.

## Results

### Demographic and Clinical Data of the Patients

The clinical features of the patients, including age, histologic grade of the tumor, tumor size, axillary lymph node status, clinical grade (TNM stage), and histology, were shown in Table 1. Paraffin-embedded tissue samples were obtained from 70 consecutive newly diagnosed patients who underwent surgery between January 1, 2001, and December 31, 2003. The median age of the patients was 49 years (range, 29–90 years). The median follow-up time for the 70 patients was 46 months (range, 3–79 months). Most of the tumors were more than 2 cm in diameter (N = 56, 80%) and poorly differentiated in histologic grade (N = 28, 40%), and most of the patients had no lymph node metastasis at the time of surgery (N = 41, 59%). The percentage of tumors at stage 1, 2 and stage 3 at the time of diagnosis was 73% and 27%, respectively. Most of the cases were infiltrating ductal carcinoma (N = 45, 64%), and the remaining 25 cases consisted of other types of breast cancer (Table 1).

**Table 1:** Relationship between FOXO3a expression and clinicopathological features of the study population.

Criteria	No. cases (%)	Mean FOXO3a/field SD (%)	P Value
**Total**	70 (100)	31.74±19.65	
**Age (years)**			0.123
<50	40 (57)	35.69±22.14	
≥50	30 (43)	26.78±14.90	
**Histologic grade**			0.002*
Well and Moderate (1, 2)	42 (60)	37.07±17.92	
Poor (3)	28 (40)	23.20±19.78	
**Tumor size (cm)**			0.229
≤2.0	14 (20)	36.67±17.95	
>2.0	56 (80)	30.29±20.10	
**Axillary lymph node**			<0.001*
N_0_	41 (59)	40.39±17.73	
N_+_	29 (41)	19.53±15.45	
**ER**			<0.001*
Negative	37 (53)	21.04±15.77	
Positive	33 (47)	43.75±16.49	
**PR**			0.079
Negative	41 (59)	28.03±19.16	
Positive	29 (41)	37.00±19.46	
**HER2 status**			0.911
Negative	33 (47)	31.52±20.08	
Positive	37 (53)	31.95±19.54	
**TNM stage**			<0.001*
1, 2	51 (73)	37.94±18.27	
3	19 (27)	16.94±14.30	
**Histology**			0.811
Ductal	45 (64)	31.05±19.43	
Others	25 (36)	32.99±20.39	

* P<0.05 is considered significant.

### Immunohistochemical (IHC) Staining for Foxo3a in Human Breast Cancer Tissues

**Figure 1:**
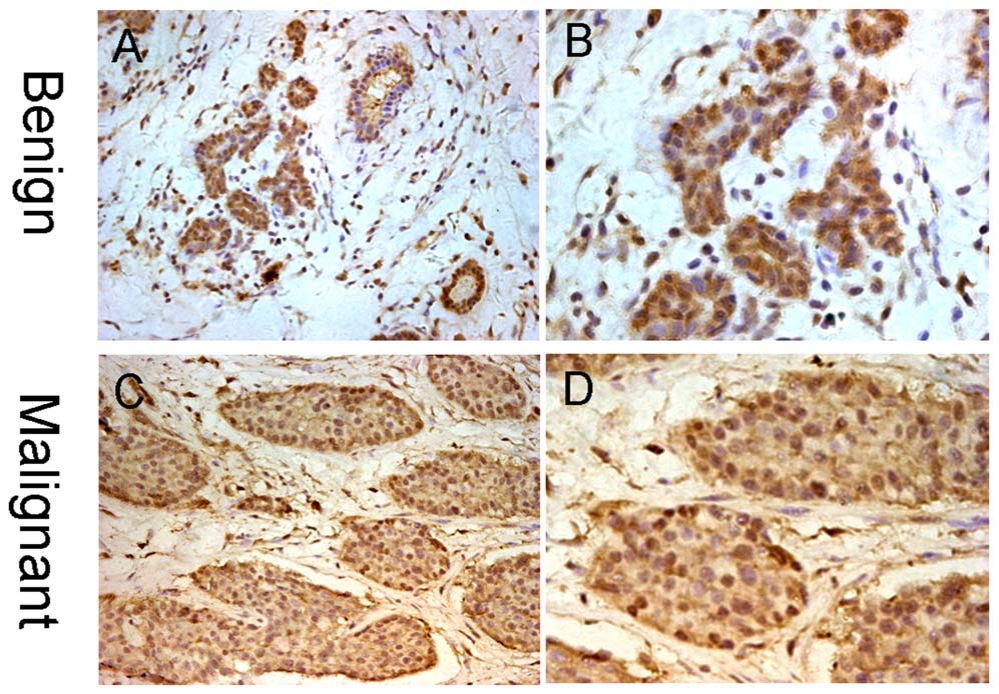
An immunohistochemical analysis of Foxo3a protein in human breast tissues. (Original magnification×200(A,C), ×400(B,D)). Expression of Foxo3a in Benign breast tissue(A,B). Brown nuclear and cytoplasmic Foxo3a staining were showed in epithelial cells lining the ducts. Expression of Foxo3a in malignant mammary gland tumors(C,D). All of the staining accumulated in the nucleus and cytoplasm.

First, IHC analysis was performed to investigate the expression of Foxo3a in paraffin-embedded human mammary tissue sections from 20 benign breast disease and 70 breast cancer patients. The Foxo3a staining was shown in Figure 1. The luminal epithelial cells from control benign breast disease and the carcinoma cells from malignant breast tumors showed brown nuclear and cytoplasmic staining for Foxo3a (Figure 1). Of the 70 tumor samples, the mean Foxo3a LI of all samples was 31.74±19.65% per case. The percentages of samples expressing low and high levels of Foxo3a were 63% (44/70) and 37% (26/70), respectively.

### Foxo3a Protein Expression Correlated with Hormone Receptor-Positive Breast Cancers

**Figure 2:**
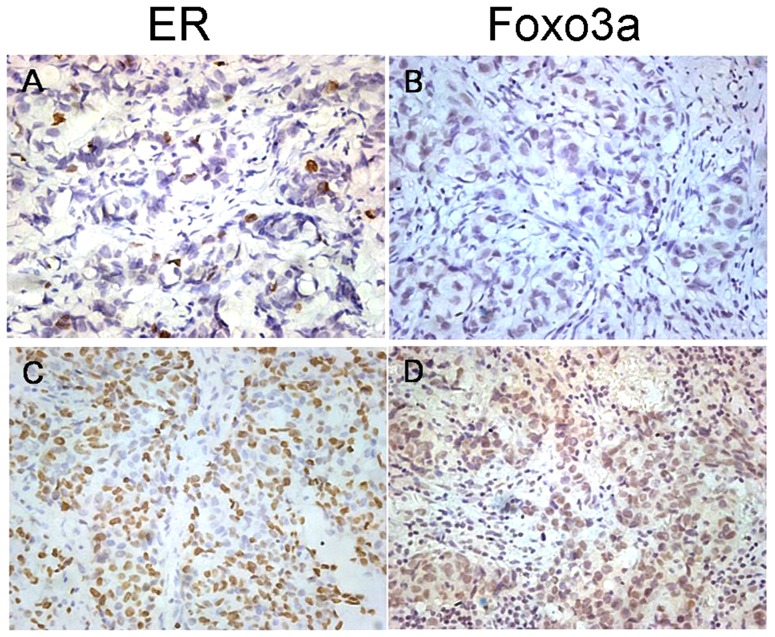
Foxo3a protein expression in distinct ER+ and ER− paraffin-embedded breast cancer tissue samples analyzed by IHC. The IHC results were shown at ×400 magnification. Expression of ER and Foxo3a in malignant mammary gland tumors. ER-negative breast cancer case (A) displayed low expression of Foxo3a (B);ER-positive breast cancer case(C) showed high expression of Foxo3a (D).

**Figure 3:**
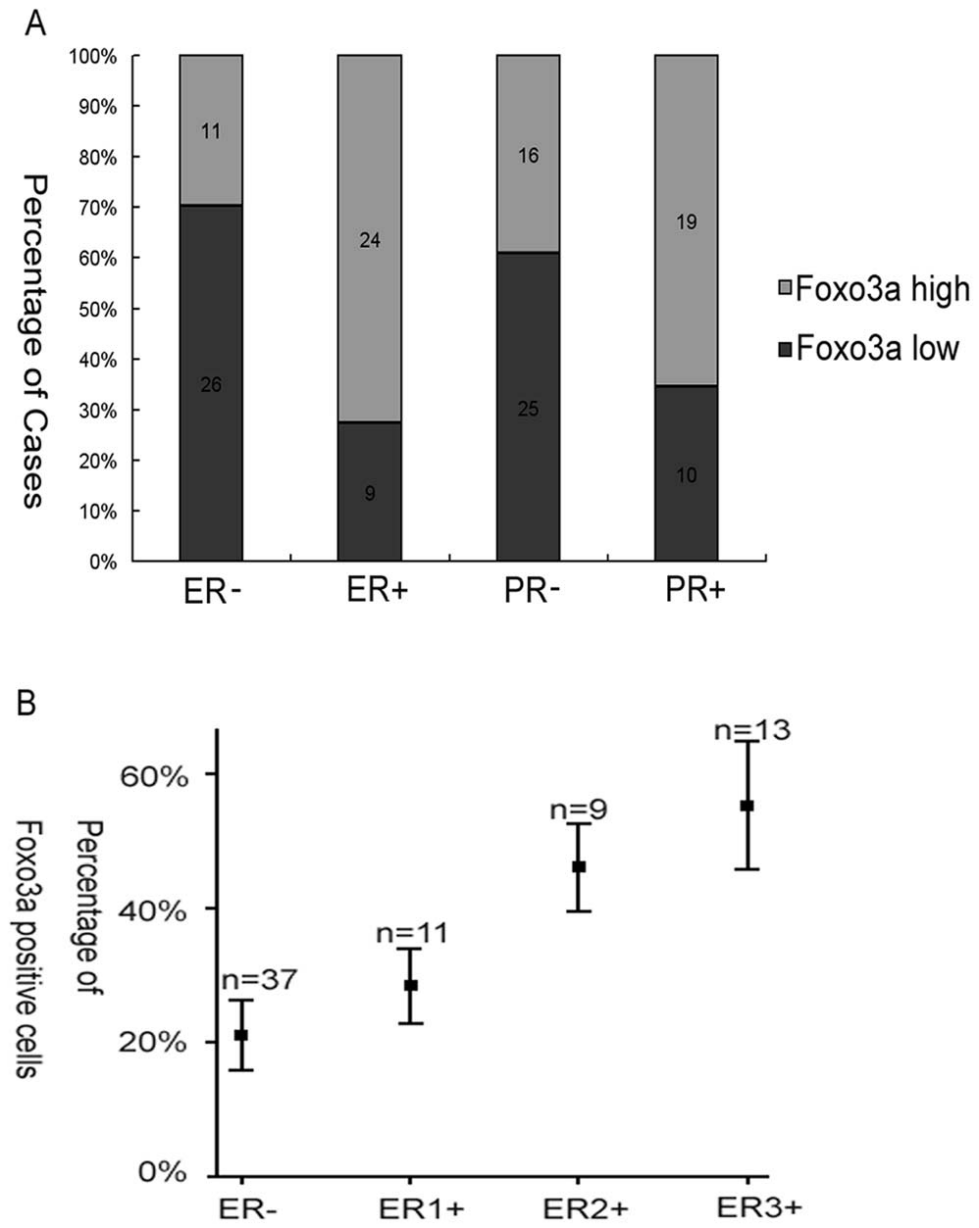
Foxo3a expression correlated with hormone receptor positivity. (A) The number of Foxo3a-positive cases was disproportionally higher in ER− positive (24) and PR-positive (19) cases compared with ER-negative (11) and PR-negative cases (16, P<0.001). (B) Similarly, there was a direct correlation between the percentage of Foxo3a-positive cells per case and the intensity of ER staining (P<0.001).

In normal breast tissue, ERa expression is observed in only 10% to 15% of luminal epithelial cells [28], which is similar to the Foxo3a expression we observed in the benign breast samples (Figure 1A). Thus, we hypothesized that the expression pattern of Foxo3a in breast tissue may be similar to ERa. To test this hypothesis, we analyzed the intensity of Foxo3a staining according to ER status. As shown in Table 1, the average Foxo3a LI was 21.04±15.77% in ER-negative tumors, which was in contrast to 43.75±16.49% in ER-positive tumors (P<0.0001). An increase in LI was also observed in PR-positive tumors compared with PR-negative tumors. Low Foxo3a expression was observed in ER-negative breast cancer samples, and ER-positive breast cancer samples had high Foxo3a expression, as shown in Figure 2. In addition, Figure 3A showed the disproportionate distribution of Foxo3a-positive tumors between hormone receptor–positive and hormone receptor–negative tumors. To further characterize the relationship between Foxo3a and the hormone receptor status, the percentage of Foxo3a-positive cells per case was plotted against the intensity of ER expression. Figure 3B demonstrates the existence of a direct relationship between the percentage of Foxo3a-positive cells and the intensity of ER expression (R = 0.663, P<0.001).

### Foxo3a Protein Expression in Distinct ER+ and ER− Human Breast Cell Lines

**Figure 4:**
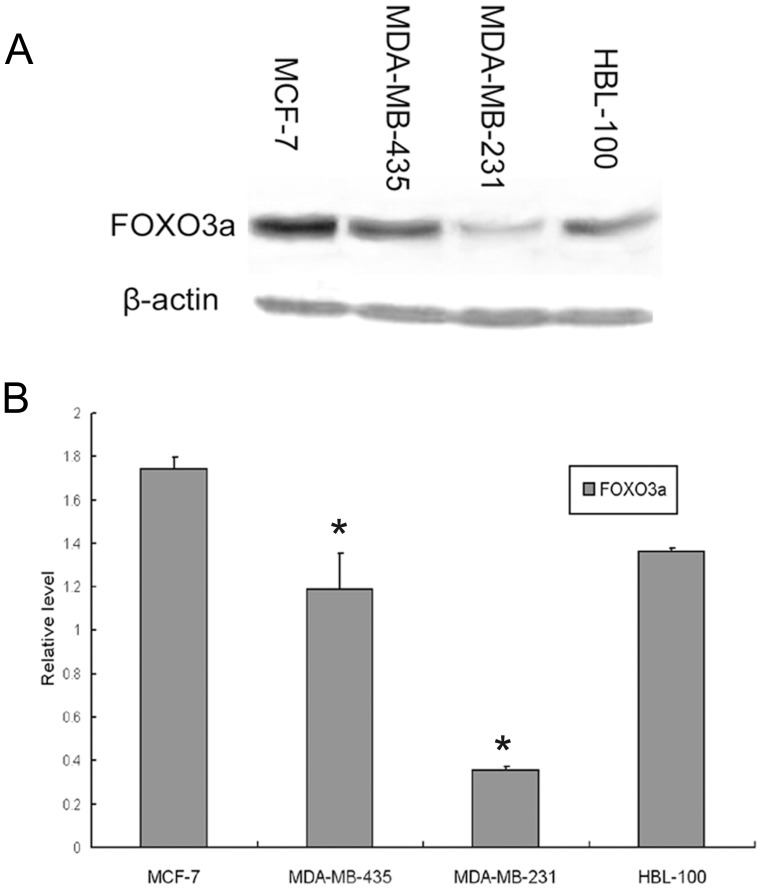
Foxo3a protein expression in distinct ER+ and ER− breast cell lines. (A) A Western blot analysis of endogenous Foxo3a abundance in three human breast cancer cell lines and one normal human breast epithelial cell line: MCF-7 (ER+), MDA-MB-435(ER−), MDA-MB-231(ER−) and HBL 100. The total protein extracted from the indicated cell lines was analyzed using a polyclonal antibody against human Foxo3a. β-actin was used as a loading control. (B) The bar graph below demonstrates the level of Foxo3a expression compared with the expression of β-actin. Error bars reflect the standard error from at least 3 independent experiments. * indicates p<0.05 for comparison between MCF-7 and MDA-MB-435 or MDA-MB-231.

The data described above prompted us to determine the basal protein expression of Foxo3a in cell lines. Thus, we investigated the relationship between Foxo3a and ER by immunohistochemical analysis in one normal human breast cell line HBL-100 and three different breast cancer cell lines, MCF-7 (ER+), MDA-MB-231 (ER−) and MDA-MB-435 (ER−). Recent studies have reported that FOXO3a suppresses estrogen-dependent breast cancer cell proliferation and tumorigenesis, and crosstalk between the Foxo3a and ER signaling pathways has been demonstrated by several laboratories [31]–[34]. In accordance with previous studies, Western blot analysis of endogenous Foxo3a in established mammary epithelial cell lines indicated that the level of Foxo3a in the ER-positive breast cancer cell line (MCF-7) was significantly higher than the two ER-negative cell lines (MDA-MB-231 and MDA-MB-435), as shown in Figure 4A, B. The cell line HBL-100, which was derived from normal mammary tissue, also showed moderate expression of Foxo3a. Collectively, these data suggest that the expression of Foxo3a might be a frequent event in human breast cancer and that the level of Foxo3a expression is correlated with ER status.

### Correlation between Foxo3a Expression and Other Clinicopathological Factors in Breast Tumors

As shown in Table 1, we compared Foxo3a expression with age histologic grade, tumor size, axillary lymph node status, ER status, PR status, HER-2 status, TNM stage and histology. Foxo3a expression was strongly associated with axillary lymph node status (P<0.001), ER status (P<0.001) and TNM stage (P<0.001). An association was also observed between Foxo3a and histologic grade (P = 0.002). In contrast, there was no significant correlation between Foxo3a and age (P = 0.123), tumor size (P = 0.229), PR status (P = 0.079), HER-2 status (P = 0.911) or histology (P = 0.811). Foxo3a expression in poorly differentiated tumors was lower than in moderately and well-differentiated tumors (P = 0.002). Moreover, the analysis revealed an inverse correlation between Foxo3a and lymph node status: a higher LI average was found in N_0_ (40.39±17.73%) tumors compared with N_+_ (19.53±15.45%) tumors (P<0.001). Similarly, patients with stage 1 and 2 tumors had higher Foxo3a expression compared with patients with stage 3 tumors.

### Positive Foxo3a Expression is an Independent Prognostic Marker of Overall Survival in Breast Cancer

**Figure 5:**
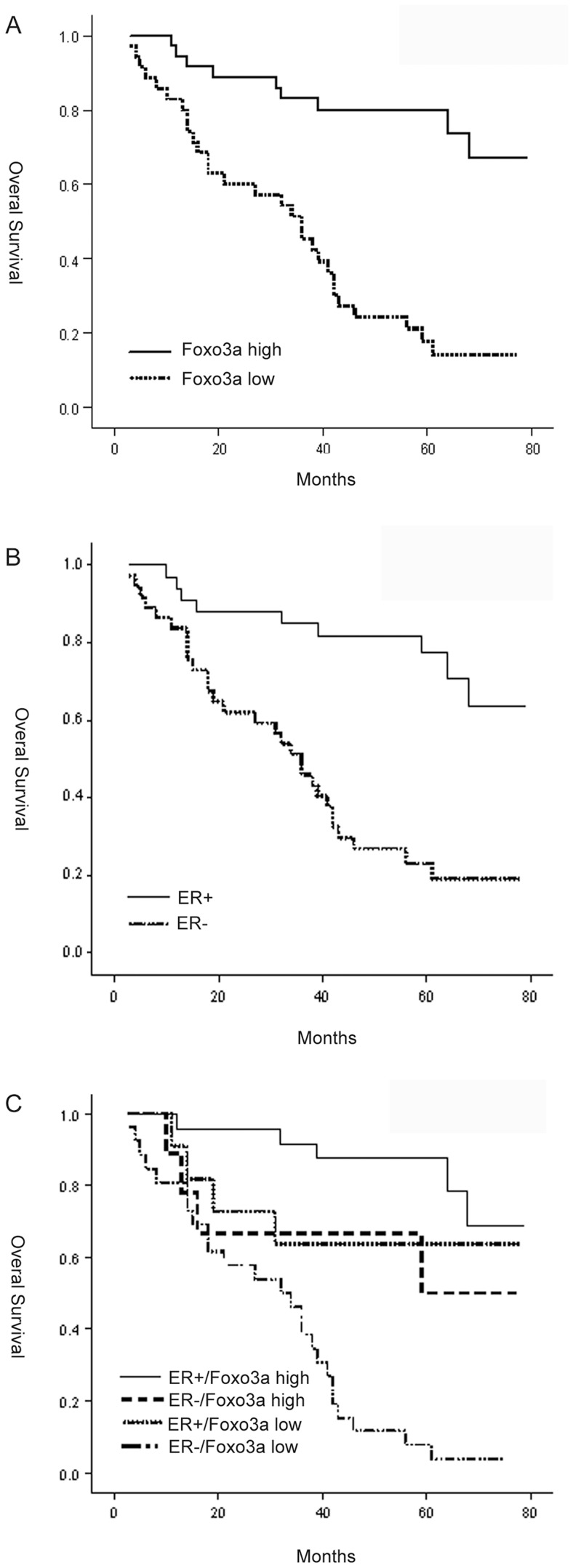
Kaplan–Meier analysis of breast cancer patients. The effect of Foxo3a (A) or ER (B) expression on overall survival in 70 patients with breast carcinoma. Kaplan-Meier curves showing the overall survival for subgroups of patients stratified by ER/Foxo3a staining (C).

We carried out a Kaplan-Meier analysis to study the correlation between Foxo3a and patient survival. Based on IHC positivity, the tumors were divided into two groups: Foxo3a-high tumors (LI≥31.7%) and Foxo3a-low tumors (LI<31.7%). The mean overall survival for patients with Foxo3a-high tumors was 66.01 months versus 35.15 months for patients with Foxo3a-low tumors (P<0.001). The patients with Foxo3a-high tumors showed increased survival compared with patients with Foxo3a-low tumors (P<0.001, Figure 5A). In accordance with a previous report, the loss of ER expression was correlated with an elevated risk of death (P<0.001, Figure 5B). Most importantly, Foxo3a-high tumors were correlated with significantly better overall survival in ER+ cases (P<0.001, Figure 5C), whereas for patients with Foxo3a-low tumors, the loss of ER was associated with a significantly worse overall survival (P = 0.013, Figure 5C). Finally, patients with ER−/Foxo3a-high and ER+/Foxo3a-low cases had intermediate survival time.

We also performed a univariate analysis of survival using a Cox proportional hazards regression model including several clinicopathological parameters (Table 2). A univariate analysis demonstrated that Foxo3a expression (P<0.0001), histologic grade (P = 0.003), tumor size (P = 0.002), axiliary lymph node status (P<0.0001) and ER status (P<0.0001) were able to predict overall survival in breast cancer. We then selected Foxo3a, ER status, histologic grade, tumor size and axiliary lymph node status for the multivariate analysis. As shown in Table 2, even when ER (P = 0.006) and TNM stage (P = 0.020) were included, demonstrating the independent Foxo3a expression (P = 0.038) was statistically significant. whereas histologic grade (P = 0.894), tumor size (P = 0.449), axillary lymph node status (P = 0.569) were insignificant in the multivariate analysis. Thus, we concluded that Foxo3a is a favorable prognostic marker of overall survival in breast cancer patients.

**Table 2:** Prognostic factors in 70 breast cancer patients compared with overall survival.

	Univariate		Multivariate	
	P	aRR (95% bCI)	P	aRR (95% bCI)
Age	0.609	1.182		
		(0.623–2.244)		
Histologic grade	0.003*	2.022	0.894	0.950
		(1.270–3.218)		(0.451–2.003)
Tumor size	0.002*	1.869	0.449	0.755
		(1.260–2.771)		(0.365–1.562)
Axillary lymph node	<0.0001*	2.282	0.569	0.795
status		(1.636–3.182)		(0.360–1.752)
ER	<0.0001*	0.215	0.006*	0.262
		(0.101–0.460)		(0.100–0.683)
HER2	0.863	0.946		
		(0.500–1.789)		
TNM stage	<0.0001*	3.591	0.020*	5.451
		(2.124–6.070)		(1.303–22.810)
Histology	0.712	0.819		
		(0.443–1.743)		
Foxo3a	<0.0001*	0.181	0.038*	0.368
		(0.085–0.387)		(0.143–0.947)

a RR, relative risk;

b CI, confidence interval.

* P<0.05 is considered significant.

## Discussion

Breast cancer is a group of heterogeneous diseases with various biological and clinical characteristics. Its incidence is increasing in many countries. Patient management is currently based on easily identifiable clinical and pathological characteristics, which only partially reflect disease heterogeneity [38]. Although standard predictive factors, including age, tumor size, histological type, axillary node involvement, histological or nuclear grade (Elston's method), and steroid receptor expression have been reported [39], it is still urgent to identify clinically useful, readily available prognostic and predictive markers in the management of breast cancer. Therefore, we conducted immunohistochemical analyses to examine Foxo3a expression in 70 breast cancer patients and compared its association with clinical significance and prognosis.

Forkhead box O (FOXO) transcription factors belong to the class of winged helix group of transcription factors. FOXO proteins have been implicated in the control of genes involved in multiple cellular processes, including the cell cycle [5], [6], cell death [7], [8], neoplastic transformation [9]–[11], epithelial-to-mesenchymal transition [12], longevity [13], [14], metabolism [15], [16], and protection from oxidative stress [17]–[19]. In addition, FOXOs are negatively regulated by the phosphoinositide-3 kinase (PI3K)/protein kinase B (PKB) pathway. FOXOs are phosphorylated by Akt on highly conserved serine and threonine residues, resulting in impaired DNA-binding activity and increased binding to the chaperone protein 14-3-3. In turn, phosphorylated Foxo proteins associate with the 14-3-3 protein, which functions as a scaffold within the cytoplasm, and are sequestered within the cytosol, rendering them unable to bind to the promoters of their target genes in the nucleus to regulate their transcription [20]. In brief, the inactivation of FOXOs controls diverse functions, including cell differentiation, proliferation, cell death, metabolism, and longevity [24].

The present study examined the correlation between Foxo3a expression and clinical response. Foxo3a protein expression correlated with ER positivity, histologic grade, axillary lymph node negativity and TNM stage. Hormone receptor–negative breast cancers traditionally have a worse prognosis and fewer available treatment options due to the ineffectiveness of hormonal therapy compared with hormone receptor–positive tumors [40]–[42]. In fact, ER plays a critical role in the growth, proliferation, and differentiation of the mammary epithelium. Recent studies have shown crosstalk between Foxo3a and ER signaling pathways [32]–[34]. One study demonstrated that the functional interaction between FOXO3a and ER plays a critical role in suppressing estrogen-dependent breast cancer cell growth by decreasing the expression of several ER-regulated genes, some of which play important roles in cell proliferation and tumorigenesis in vivo [35]. The optimum expression of these estrogen-regulated genes may occur only in cells that co-express ER and Foxo3a, and only these cells may be favored in estrogen-dependent survival and proliferation signaling pathways. Thus, Foxo3a signaling also affects ER signaling pathways, and endocrine therapy may affect both Foxo3a and ER signaling pathways. These findings, along with our data (Figures 2–5), suggest a clear link between the expression of Foxo3a and positive ER staining and increased survival in human breast cancers. This hypothesis was further supported through continued survival analysis.

For practical purposes, it is important to examine whether Foxo3a has any clinical value in predicting breast cancer progression. In our studies, a Kaplan-Meier analysis was carried out to study the correlation between Foxo3a and patient survival. We observed that patients with Foxo3a-high tumors showed increased overall survival compared with patients with Foxo3a-low tumors. As we expected, when stratified by ER status, patients who were ER+/Foxo3a+ had better prognoses than patients who were ER−/Foxo3a+. On the contrary, in Foxo3a-low cases, loss of ER was associated with significantly worse overall survival compared with ER+ cases. Thus, the simultaneous analysis of ERα and Foxo3a may provide the earliest indication for hormone independence and/or ERα: growth factor and signaling pathway cross-talk in ERα-positive breast cancers. We also carried out a univariate and multivariate analysis of survival using the Cox proportional hazards regression model. In the multivariate analysis, we found Foxo3a to be a favorable independent prognostic marker in breast cancer (RR, 0.181). Therefore, Foxo3a may serve as a significant prognostic marker for long-term survival in breast cancer. Consistent with our findings, a very similar work performed with the same polyclonal antibody by Habashy et al [43] showed that FOXO3a nuclear localisation is associated with good prognosis in luminal-like breast cancer. However, controversial data was shown by Chen et al [44]. These authors demonstrated that nuclear Foxo3a expression was associated with lymph node positivity and poor prognosis in breast cancer. Thus, the exact biological significance remains unclear and further investigations are still needed to clarify their potential role in breast carcinogenesis.

In conclusion, Foxo3a expression is an intriguing prognostic factor in breast cancer. In particular, patients with ER-positive tumors have a better overall survival. In addition, Foxo3a staining could potentially be used for patient stratification in conjunction with other prognostic markers. Thus, the data presented in this study may provide a new direction in the research of breast cancer and an opportunity for the development of potential therapy.
